# Clinical pharmacy undergraduate education in China: a comparative analysis based on ten universities’ training programs

**DOI:** 10.1186/s12909-023-04049-y

**Published:** 2023-02-02

**Authors:** Jiakai Li, Chenlin Xiao, Jingjing Hou, Yichang Zhao, Hui Gong, Bikui Zhang, Miao Yan

**Affiliations:** 1 Department of Pharmacy, The Second Xiangya Hospital of Central South University, Changsha, China; 2 Institute of Clinical Pharmacy, Central South University, Changsha, China; 3 Xiangya Schools of Pharmaceutical Sciences, Central South University, Changsha, China

**Keywords:** Clinical pharmacy, Education, Undergraduate education, China

## Abstract

**Background:**

In recent years, the scale of personnel training for clinical pharmacy professionals in China has expanded increasingly, however, the shortage of clinical pharmacists is still prominent. In 2018, the Ministry of Education of China released *national standards for the teaching quality of undergraduate majors at regular colleges and universities*, which has developed a core policy for undergraduate clinical pharmacy training. To explore the training methods for clinical pharmacy professionals in China and to promote the healthy and sustainable development of the clinical pharmacy education system. This study comparatively analyzed the training programs for clinical pharmacy undergraduates in China’s ten universities, discussed training programs suitable for clinical pharmacy professionals in China.

**Methods:**

The clinical pharmacy education programs in these ten universities were obtained through official school websites or by interviewing relevant people, and then compared and analyzed.

**Results:**

The school with the largest number of courses and the most class hours in general courses is University A1 (34 courses, 1316 class hours), and the school with the most credits is University B1 (75.5 credits). The schools with the largest number of courses and the most class hours in the basic courses are University A1 (50 courses, 1997 class hours), and the schools with the most credits are University B3 and University B1 (105.5 credits). The schools with the largest number of courses in the core courses are University C1 (23 courses), and the school with the most credits and class hours is University B2 (51 credits, 914 class hours). The school with the most class hours in practical teaching is University B6 (1406 class hours), and the schools with the longest internship time are University A1 and University B6 (52 weeks).

**Conclusions:**

There was substantial variation in programs. There remains a gap between the existing educational model and clinical training in pharmacy in China and developed countries. China should explore the most appropriate method for undergraduate education in clinical pharmacy based on studying foreign excellent educational models and the experience of China.

## Background

The American College of Clinical Pharmacy (ACCP) defines clinical pharmacy as an area of pharmacy concerned with the science and practice of rational medication use [1]. And the European Society of Clinical Pharmacy (ESCP) defines clinical pharmacy as a practice encompasses cognitive, managerial and interpersonal activities targeting all stages of the medicines use process, and as a field of research generates knowledge that informs clinical decision-making, health care organization or policy, aims to optimize the utilization of medicines in order to achieve person-centered and public health goals [2]. In China, we define clinical pharmacy is a branch of pharmacy that combines medicine and pharmacy, which discusses the law of clinical application of drugs and focuses on the rational clinical application of drugs. With the reform and development of China's medical and health services, clinical pharmacists, like doctors and nurses, have become an important part of modern medical teams. The goal of clinical pharmacy specialty is to cultivate clinical pharmacy professionals who can effectively meet the needs of clinical diagnosis and treatment services and drug guidance in the new era. Clinical pharmacy education is the main way to train clinical pharmacists, which is an important guarantee of clinical drug use safety and effectiveness.

Hospital pharmacy practice is changing from ‘‘drug-centered’’ to ‘‘patient-centered,’’ and the primary focus for hospital pharmacy is changing from drug supply to pharmaceutical care [3]. Thus, the role of clinical pharmacist is shifting from drug dispensing and compounding to rational drug use and patient care. Both clinical pharmacy education and clinical pharmacist training should be aimed at “patient-centered, rational drug use as the core.” In 2011, the former Ministry of Health issued the *Regulations of Pharmacy Affairs for Medical Institutions*, which stipulated that, the number of clinical pharmacists in tertiary hospitals shall not be less than 5, and that in secondary hospitals shall not be less than 3 [4]. This represents a large shortage in clinical pharmacists in China. It is urgent to promote the high-quality training of professional clinical pharmacists and vigorously promote the education of clinical pharmacy in China.

In 1987, The State Education Commission of the People’s Republic of China (Ministry of Education of the People’s Republic of China, MOE) listed clinical pharmacy as a new major topic for the first time. The same year, West China School of Pharmacy, Sichuan University established the first undergraduate program in clinical pharmacy in China, and formally enrolled students in 1989, offering the first 5-year Bachelor of Science degree in clinical pharmacy(Standard pharmacy with a 4-year Bachelor of Science degree) [5]. Since then, clinical pharmacy education in China has begun. Dalian Medical University in 1993 and Shenyang Pharmaceutical University in 1995 both offered Bachelor of Science degree in clinical pharmacy. However, MOE abolished the clinical pharmacy program in 1998. But the development of clinical pharmacy education did not stop. From 2006 to 2011, MOE approved 17 medical universities [6], including China Pharmaceutical University, Harbin Medical University, Shenyang Pharmaceutical University, and Anhui Medical University, etc., to establish a 5-year clinical pharmacy undergraduate program without adjusting the major catalog. In 2012, MOE relisted clinical pharmacy majors in the *undergraduate major catalog of ordinary colleges and universities*. In 2018, MOE issued the *national standards for the teaching quality of undergraduate majors in ordinary colleges and universities,* which is the first national standard for teaching quality in clinical pharmacy. It provides a 5-year program in clinical pharmacy leading to a Bachelor of Science degree upon graduation. From then on, the number of colleges and universities offering clinical pharmacy has grown rapidly in China. As of 2019, there are 498 colleges and universities offering drug-related undergraduate majors, including 52 full-time colleges and universities offering clinical pharmacy undergraduate majors [6].

The aim of this study is to explore the similarities and differences between undergraduate clinical pharmacy education programs in Chinese universities. Our research seeks to understand clinical pharmacy education to facilitate direct communication and shared advancement among educators in China. To achieve the ultimate goal of ensuring the safe, efficient, rational and cost-effective use of medications.

## Materials and methods

### Study design and data collection

Our research team includes three experts with senior educational experience in the field of clinical pharmacy and four researchers involved in clinical pharmacy education research. In addition, several experts with senior educational experience were consulted, and expert opinions were obtained. Three types of representative universities, including comprehensive universities (ranked in the top 30 in China), medical universities, and pharmaceutical universities, were selected for the study through a group discussion. In addition, based on the stratification of universities, they distributed in different regions of China were selected to send questionnaires by email or message. The questionnaire consisted of asking the respondents for information about the training programs in their schools. In summary, there were no geographical differences in the sampling method.

Data were available from the training programs of undergraduate clinical pharmacy students enrolled in 2016–2018.Each school consulted 1–3 relevant people such as teachers or students. Their information on the training programs was obtained from official documents on the school's websites, in order to guarantee the authenticity and accuracy of the documents.

Inclusion and exclusion criteria for the data in this study were as follows.

#### Inclusion criteria

1. Offering a five-year undergraduate clinical pharmacy program
2. Belonging to three types of universities, including comprehensive universities, medical universities, or pharmacy universities
3. Containing data on complete five-year clinical pharmacy training programs in any year from 2016–2018.

#### Exclusion criteria

1. Unwilling to provide information on training programs
2. Too much missing data (> 30%) for the collected training programs.

Because it is difficult to analyze the data of all colleges and universities that offer undergraduate programs in clinical pharmacy, and many colleges and universities have similarities in their programs, we sampled schools at three levels. It includes both universities that first started clinical pharmacy programs and universities that have newly started clinical pharmacy programs in recent years. The selected universities are widely distributed across the country, including the northeast, central, southeast coastal, and western regions. The coverage is wide and representative.

This study collected the undergraduate training programs of clinical pharmacy from 10 universities, including:

1. Comprehensive universities: University A1 and University A2. (A comprehensive university is a university that includes multiple disciplines and interdisciplinary fields of academic knowledge, usually with multiple colleges)
2. Medical Universities: University B1, University B2, University B3, University B4, University B5, and University B6.
3. Pharmaceutical Universities: University C1 and University C2.

The training program includes the allocation of courses, class hours, and credits for each semester.

### Curriculum

The *National Standards for the teaching quality of undergraduates in ordinary colleges and universities* divided the curriculum into four parts:

1. General curricula: including ideological and political courses, English, physical education, advanced mathematics, computer courses, employment guidance courses and other courses.
2. Basic curricula: including inorganic chemistry, organic chemistry, biochemistry, and molecular biology, analytical chemistry, pharmaceutical analysis, physiology, pathology, pathophysiology, systematic anatomy, histology and embryology, medical genetics, medical microbiology, medical immunology, diagnostics, internal medicine, surgery, obstetrics and gynecology, pediatrics, neurology, medical ethics, and other courses.
3. Core curricula: including pharmacology, medicinal chemistry, pharmacy, clinical pharmacology, clinical pharmacokinetics, clinical pharmacotherapy, pharmacogenomics, clinical pharmacy English, drug toxicology and other courses.
4. Practical teaching part: including experimental work, special discussions, case analysis, and other courses in chemistry, biology, basic medicine, and pharmacy. Some of them are separate seminar courses, others are integrated into the basic curricula and core curricula. It also recommends 180–200 credits for the five-year undergraduate curricula of clinical pharmacy and allows colleges and universities to adjust according to the current situation. In this way, the majority of clinical pharmacy major not only has the rigid baseline requirements of national standards but also has a flexible space for self-adjustment.

Based on the above curriculum classification, this study will analyze the courses, class hours, and credits allocation in ten universities, and explore the most suitable training plan for clinical pharmacy majors.

## Results

### General curricula

**Fig. 1:**
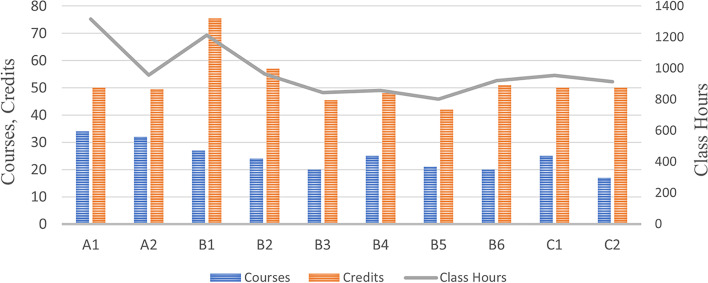
Number of courses, credits, and class hours of general curricula in ten universities

Among the ten universities, the school with the largest number of general curricula is University A1, with 34 courses, followed by University A2, with 32 courses. It is worth noting that both universities are comprehensive universities. The school with the most credits is University B1, with 75.5 credits, followed by University B2, with 57 credits. And the school with the most class hours is University A1, with 1316 class hours, followed by University B1, with 1212 class hours (Fig. 1).

### Basic curricula

**Fig. 2:**
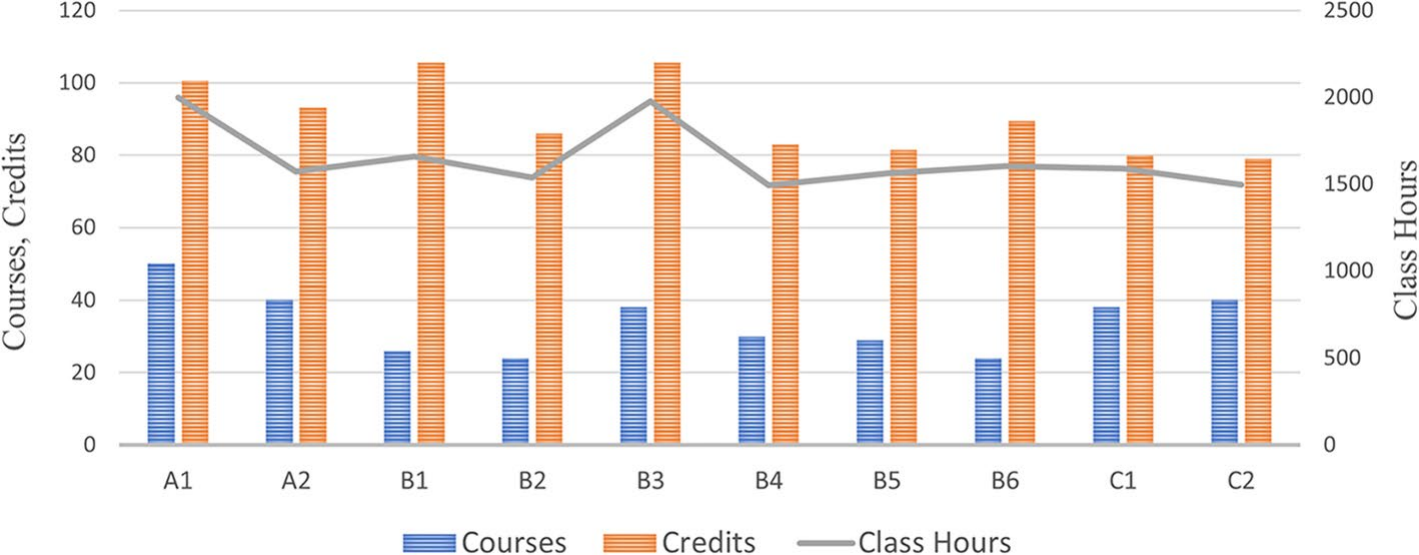
Number of courses, credits, and class hours of basic curricula in ten universities

Among the ten universities, the school with the largest number of basic curricula is University A1, with 50 courses, followed by University A2 and University C2, both with 40 courses. The school with the most credits is University B3 and University B1, both with 105.5 credits, followed by University A1, with 100.5 credits. And the school with the most class hours is University A1, with 1997 class hours, followed by University B3, with 1978 class hours. Figure 2 and we also counted the courses offered by ten universities in basic curricula and the average credits and class hours. We can find that all universities offer chemistry courses such as organic chemistry, inorganic chemistry, and medical courses such as systematic anatomy, physiology, pathology, and other subjects. However, few universities offer courses such as health law, evidence-based pharmacology nutrition, neurology, etc. Table 1 (Page 23–24).

**Table 1:** Basic curricula offered by ten universities

**Course**	**Number of universities offering this course**	**Average credit**	**Average Class Hour**
Organic Chemistry	10	6.1	116.8
Inorganic Chemistry	10	5.5	105.7
Biochemistry And Molecular Biology	10	5.5	102.7
Systematic Anatomy	10	4.2	73.8
Medical Statistics	10	2.9	50.1
Medicine Analysis	10	7.4	91.2
Physiology	10	4.1	73.0
Pathophysiology	10	2.7	52.0
Diagnostics	10	4.3	79.5
Medical Microbiology	10	2.3	42.7
Medical Immunology	10	2.5	46.9
Obstetrics And Gynecology	9	2.2	38.8
Pediatrics	9	2.1	36.7
Medical Ethics	9	1.6	27.1
Medical Cell Biology	8	2.3	42.6
Analytical Chemistry	8	5.6	105.3
Pathology	8	3.6	62.8
Internal Medicine	8	6.4	111.9
Surgery	8	3.1	52.6
Pharmaceutical Economics	8	2.1	36.3
Traditional Chinese Medicine	7	2.6	44.4
Doctor-Patient Communication	7	1.3	21.9
Epidemiology	7	2.2	38.0
Medical Psychology	6	1.8	27.7
Histology And Embryology	5	2.8	50.4
Infectious Diseases	5	2.3	38.6
Evidence-Based Medicine	4	1.1	19.0
Pharmacognosy	4	2.8	52.8
Medical Genetics	3	1.8	31.0
Neurology	3	2.0	37.7
Nutrition	3	1.7	27.3
Physical Chemistry	2	4.5	80.0
Medical Imaging	2	2.5	40.0
Drug Design	2	1.8	29.5
Pharmaceutical Information	2	1.8	32.0
Medical Physics	1	6.0	112.0
Emergency Medicine	1	1.0	16.0
Rehabilitation Medicine	1	1.0	16.0
Pharmaceutical Preparations	1	1.5	27.0
Health Law	1	1.0	16.0
Evidence-Based Pharmacy	1	2.0	32.0
Systematic Integration of Clinical Curriculum^a^	1	8.0	160.0

^a^Systematic integration of clinical courses including internal medicine, surgery, etc.

### Core curricula

**Fig. 3:**
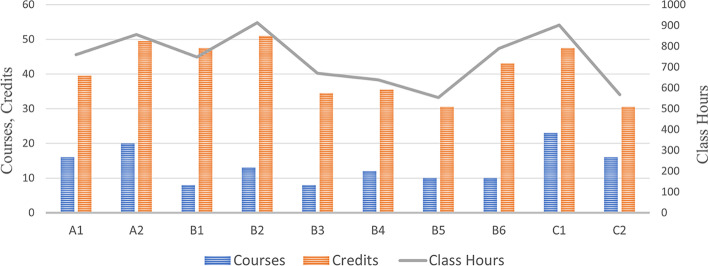
Number of courses, credits, and class hours of core curricula in ten universities

Among the ten universities, the school with the largest number of core curricula is University C1, with 23 courses, followed by University A2, with 20 courses. The school with the most credits is University B2, with 51 credits, followed by University A2, with 49.5 credits. And the school with the most class hours is University B2, with 914 class hours, followed by University C1, with 902 class hours (Fig. 3).

We also counted the courses offered by ten universities in core curricula and the average credits and class hours. We can find that all universities offer subjects such as pharmacology, pharmacy, pharmacy administration, clinical pharmacotherapy, etc. However, courses such as genetic pharmacology, pharmacovigilance, pharmacogenetic diseases, pharmacovigilance, are only offered by very few universities (Table 2).

**Table 2:** Core curricula offered by ten universities

**Course**	**Number of universities offering this course**	**Average credit**	**Average Class Hour**
Pharmacy Administration	10	2.8	46.5
Pharmaceutics	10	5.2	101.1
Pharmacokinetic	10	3.8	70.6
Medicinal Chemistry	10	4.4	78.6
Pharmacology	10	5.2	92.8
Clinical Pharmacotherapeutics	10	6.5	114.2
Clinical Pharmacology	9	3.8	69.4
Drug Toxicology	6	2.5	43.7
Clinical Pharmacy English	6	2.2	37.7
Biopharmaceuticals	5	1.7	33.6
Introduction to Clinical Pharmacy	4	1.8	31.0
Natural Products Chemistry	3	3.0	52.3
Pharmacogenetics	3	1.8	36.3
Pharmacovigilance	2	2.0	33.0
Drug-Induced Diseases and Prevention	2	1.8	28.0
Pharmacy Services	1	3.0	48.0
Drug Clinical Trials	1	3.0	51.0
Pharmaceutical Care	1	4.0	68.0

### Practical teaching part

**Fig. 4:**
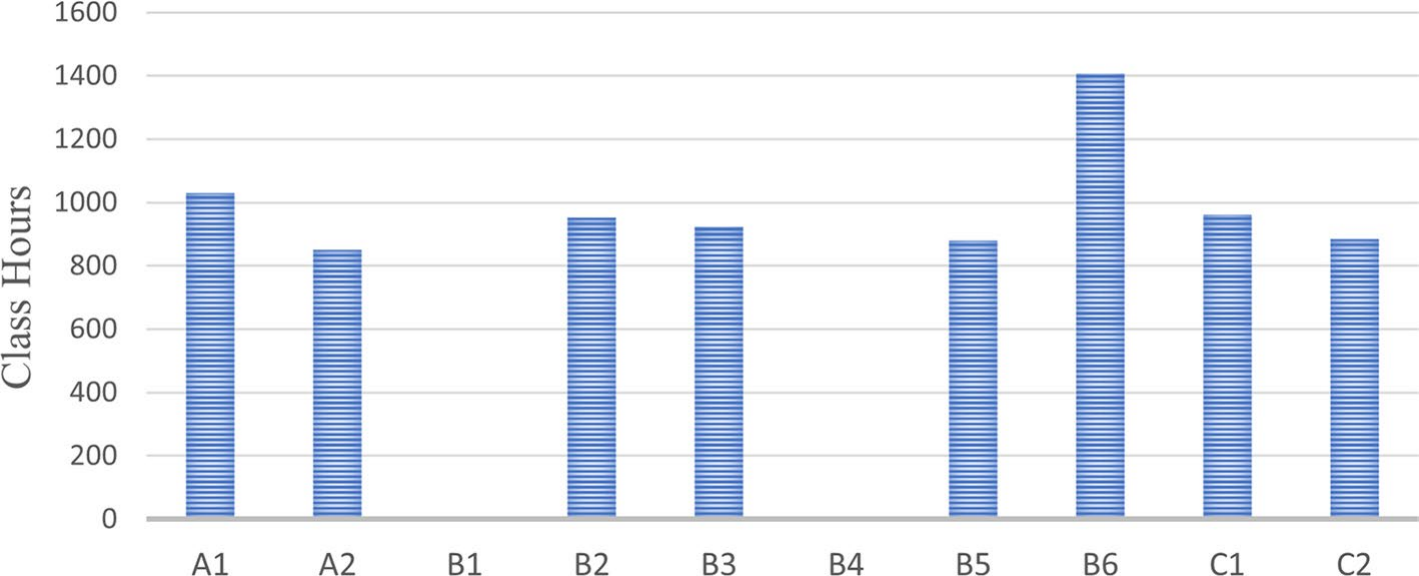
Number of class hours of practical teaching part in ten universities

This study only discusses the credit hours of practical courses and does not calculate the number of courses and credits. According to the national standards, the total class hours of practical curricula shall not be less than 600 class hours. In addition, clinical pharmacy undergraduates are also required to undertake a graduate internship of no less than 42 weeks before graduation. Among them, the internship time in the pharmacy department should not be less than 12 weeks, and the clinical internship time should not be less than 30 weeks. Among the ten universities, practice class hours from University B4 and University B1 were not available for this study. Among the other eight universities, the school with the most class hours is University B6, with 1406 class hours, followed by University A1, with 1030 class hours. The universities with the fewest class hours also have 850 class hours of practical curricula. In other words, these eight universities all meet the requirements of the national standard. Figure 4 in addition, these ten universities have set up a graduation internship of no less than 42 weeks by national standards. Among them, the graduation internship time of University A1 and University B6 even reached 52 weeks.

### Comparative analysis of ten university programs and national standards

The goal of the clinical pharmacy major is to cultivate clinical pharmacy service-oriented professionals who can effectively meet the needs of clinical diagnosis, treatment services and drug guidance in the new era, while clinical pharmacy education is the main way to train clinical pharmacists and the important guarantee for the safety and effectiveness of clinical drug use.

From the above ten universities' undergraduate training programs in clinical pharmacy, it can be seen that most of the schools' teaching contents and curricula arrangement meet the requirements of the national standards. However, different universities will offer different courses, making the courses vary from university to university. The curricula of University A2, University A1, University B4, University B6, and University C2 fully conform to national standards. In addition, University A2 systematically integrates clinical medicine courses (internal medicine, surgery, etc.) before teaching them to students, rather than stacking independent courses. Such a course arrangement can enable students to have a more systematic, comprehensive, and relevant mastery and understanding of the study of clinical medical knowledge [7]. However, there are still some universities have incomplete course offerings. For example, University B2, University B5, University B1, and University C1 lack biopharmaceutical courses. Doctor-patient communication courses in University B3, University B5, and University B1 are missing. University C1 lacks clinical medicine courses such as internal medicine, surgery, obstetrics and gynecology, and pediatrics. University B5 still lacks medical ethics courses. The emergence of these situations reminds us that it is necessary for colleges and universities that offer undergraduate clinical pharmacy majors to strictly implement national standards, conduct self-examination, and actively improve their curriculum to achieve the goal of high-quality education. This study also found that different universities offer different courses to students. Based on national standards, some universities also provide various professional courses that are conducive to students' future career development.

Among the practical teaching links, the internship links in most universities are carried out in the fourth and fifth academic years. In contrast, there are no courses related to hospital pharmacy apprenticeship or clinical apprenticeship in the lower division (first, second, and third year) curriculum. Only University C1 integrates hospital practice or community pharmacy practice into the entire teaching process. That is, from the beginning of student enrollment until graduation, there will be a corresponding internship in each academic year, and the content and difficulty of the internship are increasing. Incorporating hospital pharmacy apprenticeships or clinical apprenticeships into lower-level courses promotes students' understanding of the work of hospital pharmacists and their career plans after graduation, as well as increasing their interest in theoretical course work [8].

In general, the ten universities in this study have rich and substantial teaching contents in clinical pharmacy undergraduate courses, complete practical course structure and up to a standard number of class hours, basically meeting the requirements of national standards.

## Discussion

### Exploring curriculum programs suitable for China

Compared with developed countries, the construction of clinical pharmacy education in China is relatively late, but it is in a period of rapid development. With the introduction of national standards, colleges and universities will modify curricula based on requirements. Nevertheless, there are still many shortcomings in China's clinical pharmacy courses. For example, the proportion of chemistry courses is too large, the professional courses are simply superimposed rather than the system incorporated with the original pharmaceutical and clinical medicine courses, the cohesion between the courses is not strong [9].

Therefore, given the above gaps, Chinese universities and colleges should make changes to respond to the country's and society's needs for high quality clinical pharmacy professionals. First of all, chemistry courses should be scaled down appropriately, and basic chemistry courses should be heavily integrated and refined [10, 11]. As the main courses of traditional pharmacy majors, the importances of inorganic chemistry, organic chemistry, analytical chemistry, physical chemistry, and other chemistry courses in clinical pharmacy are very limited [12, 13]. Consequently, the number of chemistry classes should be reduced appropriately and more teaching time should be allocated to other courses. On the one hand, it can reduce school pressure on students, and on the other hand, it can teach more efficiently according to the professional requirements of clinical pharmacy. Second, universities should enhance the system of clinical medical courses. Clinical medicine courses are the important bridge to establish the link between drugs and disease. However, this part of the curriculum has deficiencies in the current practice of clinical pharmacy education in China [13, 14]. For example, most colleges and universities teach clinical medicine courses as an introduction or general theory. This means that students rarely understand the basic theory of disease in the learning process. Therefore, strengthening clinical medicine courses can help clinical pharmacy professionals to better understand illnesses and lay the foundation for future clinical services [15]. In addition, in the study of clinical courses, students can increase communication with clinicians, nurses and patients, and practice their clinical thinking skills to communicate with clinicians and patients. Third, universities should establish a comprehensive and pragmatic clinical pharmacy courses group. Due to teacher limitations and the lack of educational experience, based on our findings, current clinical pharmacy program continues to be a weak link in the development of clinical pharmacy professionals in China. Most colleges and universities have issues like the lack of courses and the lack of close integration of pharmacy and clinical practice [16]. For this purpose, Chinese experts and academics should work together to publish more high-quality medical integration educational materials as soon as possible. In this way, the professional education of clinical pharmacy students can be more useful. For example, not only the key points of clinical medication use, but also the theoretical knowledge of clinical pharmacy students can be improved. The practice of pharmacy, also known as experiential education, aims to enable students to get out of classrooms and laboratories, into society, into the real pharmaceutical and hospital environment, convert the knowledge and skills they have learned into work experience, and cultivate students' ability to apply what they have learned, to independently judge and to solve practical problems [17]. Currently, clinical practice in most universities in China is concentrated within the fourth and fifth academic years. This arrangement will prevent students from learning about the role of clinical pharmacy in clinical practice as they enter university. Therefore, practical courses can be established from the beginning of student admission, and students can form a preliminary impression of future work by introduction, observation. One way of doing this would be through entrustable professional activities [18]. With the continual enhancement of theoretical knowledge, the content of clinical practice should also continue to be deepened until it is connected to the full internship stage of graduation [7].

### Analysis of clinical pharmacy education in the United States, Europe, and Japan

Additionally, clinical pharmacy training in China should also explore a longer education model. For example, learning the six-year Pharm.D. training method in the United States. Pharm.D. has since become the only admission degree to become a licensed pharmacist [19]. Compared to the United States, the clinical pharmacy education courses offered in China are generally the same, but the teaching methods and the modalities of the practical courses are different [20]. Problem-based learning (PBL) discussion courses are usually offered at U.S. colleges and universities. Students take leadership and cultivate independent learning capacity [9], which is conducive to the formation of independent innovative capacity of students. However, in higher education in China, educators are still mainly teaching, and this traditional teaching method needs urgent improvement. In practical courses, according to the Accreditation Council for Pharmacy Education Accreditation Standards and Guidelines, pharmacy practice experience (PPE) is divided into two phases: introductory pharmacy practice experience (IPPE), which is provided in the first 3 years of a Pharm.D. program, and advanced pharmacy practice experience (APPE), which provides students with an opportunity to practice in different healthcare settings in their final year [21]. The clinical pharmacy practice course in the United States are based on and supplemented by academic courses, the difficulty of practice gradually increases, allowing students to apply the knowledge acquired in the classroom to the practice of pharmacy, to cultivate students’ clinical skills in various practical situations [22]. In contrast, clinical pharmacy practice in Chinese universities is concentrated in the fourth and fifth academic years. ​Such a context might separate students' theoretical knowledge from their clinical experience, and students may be difficult to bring what they have learned into clinical practice into pharmacy services. As a result, we suggest that practical courses should be appropriately integrated into the curriculum of each school year, whether in a community pharmacy or in a hospital. Furthermore, students also prefer ward-based practical courses, and students' liking could in turn also bring good results to teaching [23, 24].

Europe, which is the continent with the largest number of developed countries in the world, has also received a constant focus on the development of clinical pharmacy. The development of clinical pharmacy in European hospitals, which started about 10 years after the United States, can be roughly divided into the following stages: 1) Start-up stage: 1960–1975, 2) Exploration stage: 1976–1990, 3) Development stage: 1991–2000, and 4) Maturity stage: 2001-present [25]. However, because of a large number of European countries, almost all of which have undergraduate clinical pharmacy training programs, the differences in healthcare systems in different countries and the different policies regarding clinical pharmacy education among different countries have led to a relatively slow progress in clinical pharmacy across Europe and to an uneven rate of development in various countries [26]. Given this situation, this study briefly looks at the United Kingdom as an example. There are two main types of pharmacy programs in the UK. The first is the 4-year M.Pharm program, which enables students to graduate as a pre-registered pharmacist and apply for registration as a pharmacist after one year of training. The other is the five-year M.Pharm, which includes two half-year practical training courses in the 3rd and 5th years respectively, and students can apply for registration as pharmacists directly after completing this course [27]. The General Pharmaceutical Council (GPhC), the British regulator of pharmacy professionals, has developed a unified orientation program for its 24 certified pharmacy schools, and the courses for both programs of study must comply with GPhC's regulations, which include six broad areas: patients, drug efficiency, raw materials, pharmaceuticals, health systems and pharmacy knowledge extension [11]. Higher education in the UK is primarily interdisciplinary and concentrates on combining laboratories and theoretical courses. Students participate in instructional discussions and take the initiative in the classroom to develop self-directed learning and practical skills, as well as to exercise independence and innovation skill [28]. More recently, UK Schools of pharmacy have been directed to include additional practical training, with leaders in this area utilizing entrustable professional activities to structure clinical pharmacy training safely [18].

Japan is one of the few developed Asian nations and a neighbor of China. The traditional Japanese teaching of pharmaceuticals is based on academic science. At the beginning of the twenty-first century, they began learning the American model of pharmacy education and led a six-year national reform of pharmacist-oriented pharmaceutical education [29]. And it has also become the only level of admission to become a licensed pharmacist in the future. But universities continue to nurture academic talent. It has similarities with the long-term focus of China on chemistry and less medicine in pharmaceutical education [30]. Nie Xiaoyan et al. [31] analyzed the teaching model of pharmacy at The University of Tokyo and Osaka University in Japan. Osaka University continued its reform based on the initial six-year undergraduate training in pharmacy and began a new six-year training in pharmacy and a 10-year training in medicine in pharmaceutical science. While maintaining the benefits of traditional pharmacy talent development, Osaka University is actively innovating the applied and academic education of pharmacy talent. This composite model of pharmacy skills training is worthy of reference as a reform measure for undergraduate clinical pharmacy training in China.

### Limitation

This study was conducted by using the educational training programs of ten colleges and universities, among the fifty-odd colleges and universities in China that offer undergraduate programs in clinical pharmacy. Although we believe that they are very representative, there is a potential possibility that they do not fully represent the real situation of undergraduate clinical pharmacy education in China, thus we anticipate carrying out studies with larger samples and richer courses in future studies. Furthermore, this study is a comparative study of the distribution of courses, credit hours and credits offered by ten universities on a macro level. The course names used in this study are based on national standards for the quality of undergraduate majors' education in promulgated general higher education institutions by the MOE as the standard for relevant statistics. Therefore, the results of this study may not be consistent with the actual results due to inconsistencies in courses names in some universities. We also plan to make a more microscopic and detailed comparison based on one course level in future studies.

### Prospect

Due to the late start of clinical pharmacy development in China, the current state of development is approximately equivalent to the level of the 1980s in the United States [32] and close to the European level [33]. That is, the evaluation system of clinical pharmacy is being established. Therefore, China must actively absorb foreign excellent teaching experience, find a path that is most suitable for the development of clinical pharmacy in China, promote the reform of China's clinical pharmacy training model and curriculum system, and form a development model with Chinese characteristics.

## Conclusions

Clinical pharmacy education in China is generally developing at a rapid pace. Our findings indicate wide variation in clinical pharmacy education across China. This study analyzes the undergraduate education of clinical pharmacy in ten domestic universities and puts forward some reform suggestions. Each university should also check and improve the courses set by itself against the national and international standards, and set up more characteristic professional courses with the advantages of their university disciplines. We believe that if the development of these reforms can be promoted, the development of clinical pharmacy in China will go further. To cultivate more comprehensive, refined, and professional clinical pharmacy professionals with strong professional ability, strong quality, and keeping pace with the times to meet the growing demand for clinical pharmacy services in China.

## Data Availability

The datasets used and analyzed during the current study are available from the corresponding author on reasonable request.
